# Ulcerative Rash in Brown Skin

**DOI:** 10.1016/j.acepjo.2026.100517

**Published:** 2026-10-02

**Authors:** Joy Muwanes, Lilian Kim, Jesse Kellar, Ashley Garispe

**Affiliations:** 1 Department of Emergency Medicine, Saint Agnes Medical Center, Fresno, CA, USA; 2 Department of Emergency Medicine, Adventist Health Reedley, Reedley, CA, USA

## 1 Case Presentation

A 21-year-old LatinX man with a past medical history of asthma, severe atopic dermatitis, and cold sores presented with a worsening, painful rash on his face and upper body for 3 days. He reported taking Upadacitinib daily for the past 2 years. Notably, he was febrile (39.6 **°**C) and tachycardic (112 bpm). His exam revealed a diffuse ulcerative rash with honey-crusted scabbing involving the face and upper body (Figure 1, Figure 2). Laboratory studies revealed a neutrophilic predominance (85.3%) without leukocytosis, thrombocytopenia (110 K/mcL), and mild hyponatremia (130 mmol/L) (Table 1).

**Figure 1:**
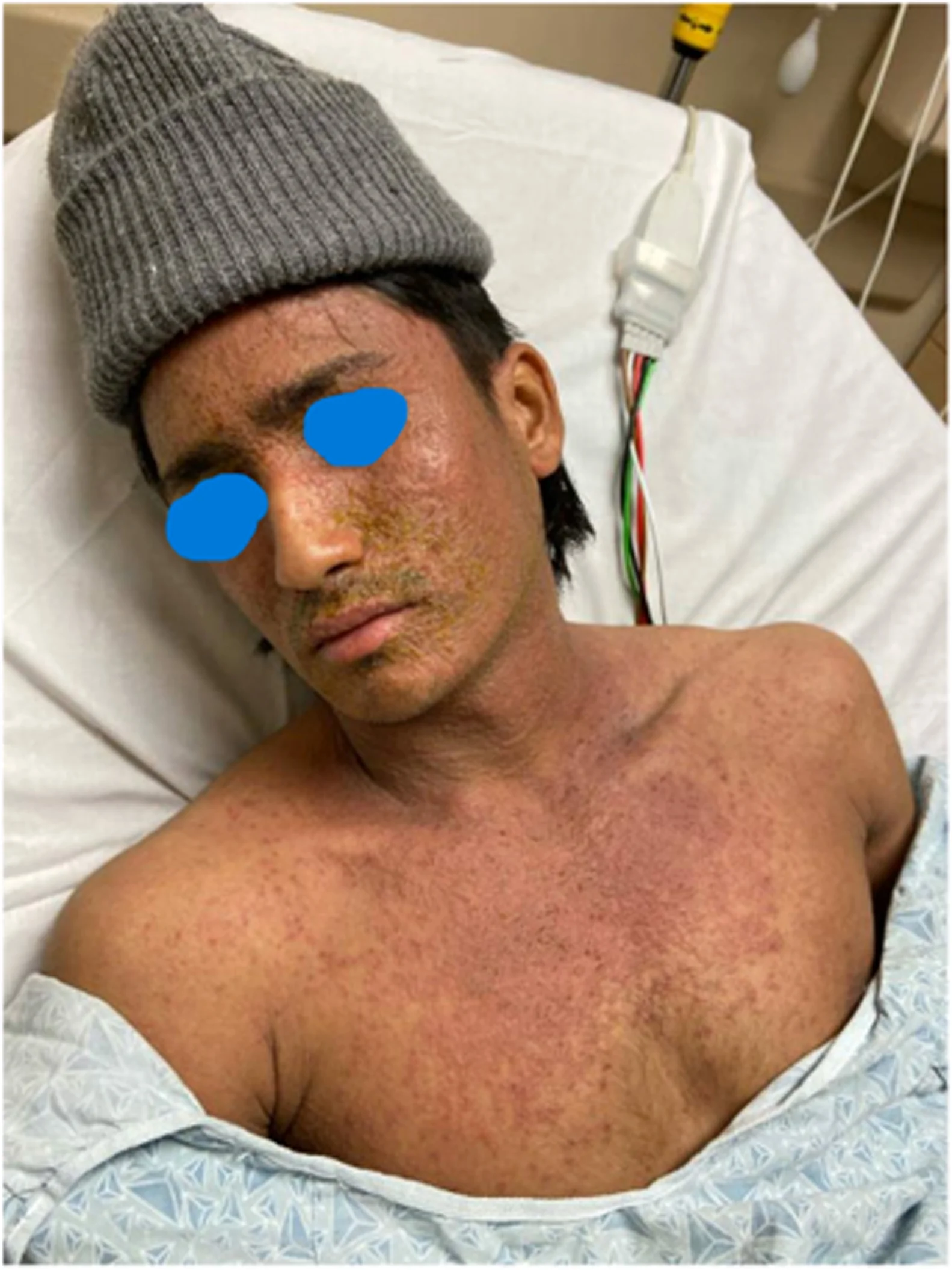
Photograph of skin lesions (day 3 of symptoms)—diffuse ulcerative rash with honey-crusted scabbing, involving the patient’s face, neck, and upper chest.

**Figure 2:**
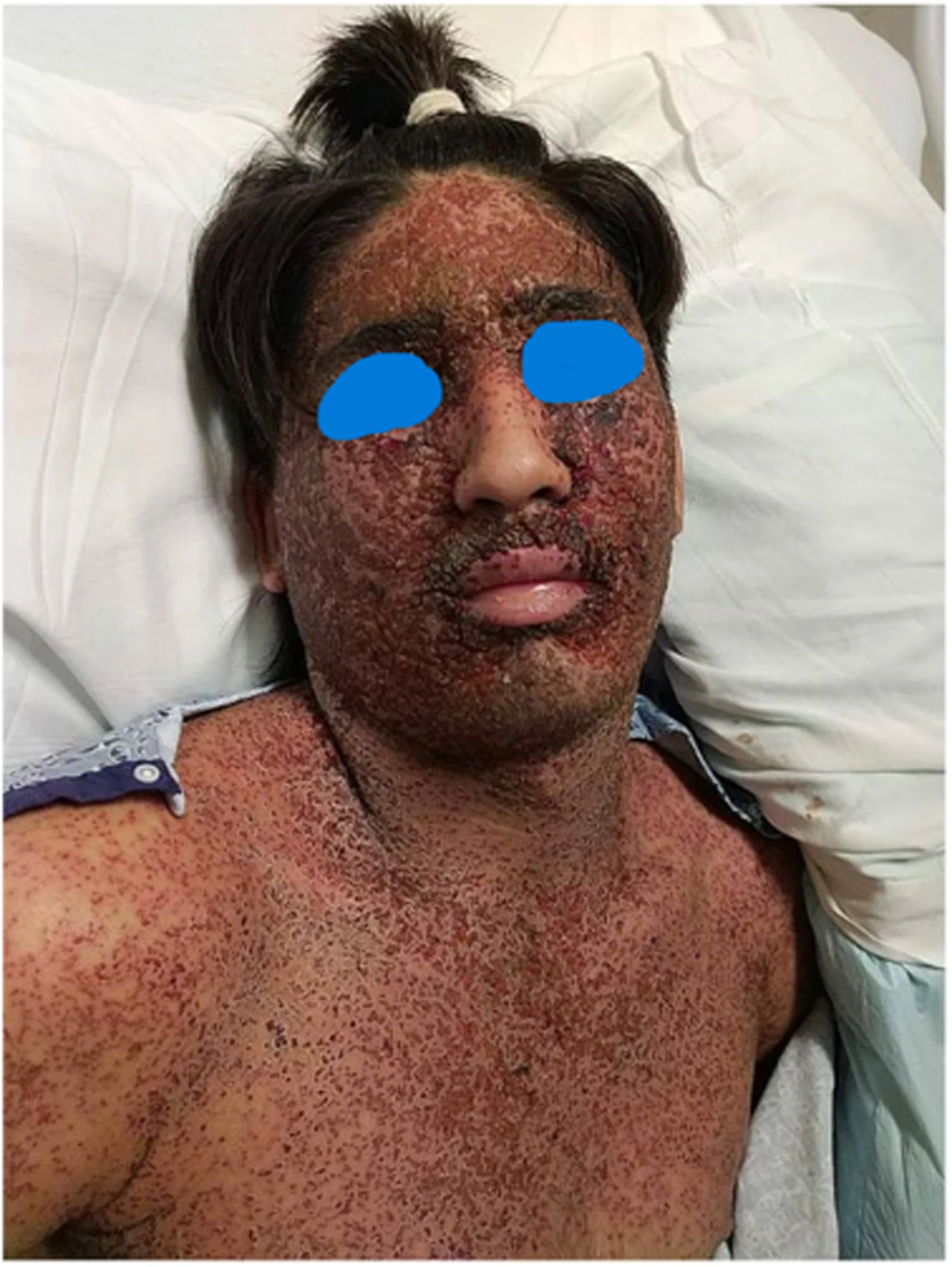
Photograph of skin lesions (day 7 of symptoms)—progression of ulcerative lesions with evidence of desquamation across the patient’s face, chest, and arms.

**Table:** Patient’s pertinent laboratory results, which demonstrate a neutrophilic predominance without leukocytosis, thrombocytopenia, and mild hyponatremia.

CBC/Differential	Results	Serum Chemistry	Results
WBC	8.5 K/mcL	Sodium	130 mmol/L
RBC	5.17 M/mcL	Potassium	3.8 mmol/L
Hemoglobin	16.5 g/dL	Chloride	95 mmol/L
Platelets	110 K/mcL	CO_2_	23 mmol/L
Neutrophils Relative	85.3%	Glucose	106 mg/dL
Lymphocytes Relative	3.6%	BUN	17 mg/dL
Neutrophils Percent Manual	Not calculated	Cr	0.91 mg/dL
Lymphocytes Percent Manual	Not calculated	AST	30 unit/L
Bands Percent Manual	Not calculated	ALT	15 unit/L
		TBili	1.4 mg/dL

CBC, complete blood count; CO_2,_ carbon dioxide; BUN, blood urea nitrogen; Cr, creatinine; AST, aspartate aminotransferase; ALT, alanine aminotransferase; Tbili, total bilirubin.

## 2 Diagnosis: Eczema Herpeticum With Superimposed Impetigo

Due to the high degree of suspicion of eczema herpeticum (EH), the patient was administered Acyclovir as well as Vancomycin, to treat the superimposed impetigo. This patient’s history and examination were consistent with EH, a serious disseminated cutaneous infection, typically caused by herpes simplex virus type 1 (HSV-1) that primarily affects patients with underlying atopic dermatitis.^1^ Although the rash occurs in less than 3% of patients with atopic dermatitis on immunosuppressive agents, EH can spread rapidly if left untreated and can result in severe morbidity and mortality of 10% to 50%.^1^ Given the critical nature of this disease, it is imperative for the treating physician to have a high index of suspicion for EH, so treatment can be initiated early.^2^ A potential limiting factor for identifying this rash is the lack of published dermatological images depicting EH lesions on patients with darker complexions.^3^ The images published in this case study intend to help clinicians identify EH in a more diverse patient population.

## Ethics declaration

Written informed consent to take part in the study and to publish the article has been obtained from all participants or their legal representatives. The privacy rights of participants have been observed.

This study was performed in compliance with relevant laws, regulatory frameworks and guidelines where the research took place. Ethics committee approval was not required under relevant laws and institutional guidelines. The patient signed a consent form for us to publish his case and image.

## Funding and Support

By *JACEP Open* policy, all authors are required to disclose any and all commercial, financial, and other relationships in any way related to the subject of this article as per ICMJE conflict of interest guidelines (see www.icmje.org). The authors have stated that no such relationships exist.

## Conflict of Interest

All authors have affirmed they have no conflicts of interest to declare.
